# Evaluating the Effectiveness and Safety of the Electroencephalogram-Based Brain-Machine Interface Rehabilitation System for Patients With Severe Hemiparetic Stroke: Protocol for a Randomized Controlled Trial (BEST-BRAIN Trial)

**DOI:** 10.2196/12339

**Published:** 2018-12-06

**Authors:** Katsuhiro Mizuno, Takayuki Abe, Junichi Ushiba, Michiyuki Kawakami, Tomomi Ohwa, Kazuto Hagimura, Miho Ogura, Kohei Okuyama, Toshiyuki Fujiwara, Meigen Liu

**Affiliations:** 1 Department of Rehabilitation Medicine Keio University School of Medicine Tokyo Japan; 2 School of Data Science Yokohama City University Yokohama Japan; 3 Keio University School of Medicine Tokyo Japan; 4 Department of Biosciences and Informatics Faculty of Science and Technology Keio University Yokohama Japan; 5 Keio University Hospital Clinical and Translational Research Center Tokyo Japan; 6 Department of Rehabilitation Medicine Juntendo University Graduate School of Medicine Tokyo Japan

**Keywords:** brain-computer interfaces, neurofeedback, neural plasticity, electroencephalography, hemiplegia, electric stimulation, robotics

## Abstract

**Background:**

We developed a brain-machine interface (BMI) system for poststroke patients with severe hemiplegia to detect event-related desynchronization (ERD) on scalp electroencephalogram (EEG) and to operate a motor-driven hand orthosis combined with neuromuscular electrical stimulation. ERD arises when the excitability of the ipsi-lesional sensorimotor cortex increases.

**Objective:**

The aim of this study was to evaluate our hypothesis that motor training using this BMI system could improve severe hemiparesis that is resistant to improvement by conventional rehabilitation. We, therefore, planned and implemented a randomized controlled clinical trial (RCT) to evaluate the effectiveness and safety of intensive rehabilitation using the BMI system.

**Methods:**

We conducted a single blind, multicenter RCT and recruited chronic poststroke patients with severe hemiparesis more than 90 days after onset (N=40). Participants were randomly allocated to the BMI group (n=20) or the control group (n=20). Patients in the BMI group repeated 10-second motor attempts to operate EEG-BMI 40 min every day followed by 40 min of conventional occupational therapy. The interventions were repeated 10 times in 2 weeks. Control participants performed a simple motor imagery without servo-action of the orthosis, and electrostimulation was given for 10 seconds for 40 min, similar to the BMI intervention. Overall, 40 min of conventional occupational therapy was also given every day after the control intervention, which was also repeated 10 times in 2 weeks. Motor functions and electrophysiological phenotypes of the paretic hands were characterized before (baseline), immediately after (post), and 4 weeks after (follow-up) the intervention. Improvement in the upper extremity score of the Fugl-Meyer assessment between baseline and follow-up was the main outcome of this study.

**Results:**

Recruitment started in March 2017 and ended in July 2018. This trial is currently in the data correcting phase. This RCT is expected to be completed by October 31, 2018.

**Conclusions:**

No widely accepted intervention has been established to improve finger function of chronic poststroke patients with severe hemiparesis. The results of this study will provide clinical data for regulatory approval and novel, important understanding of the role of sensory-motor feedback based on BMI to induce neural plasticity and motor recovery.

**Trial Registration:**

UMIN Clinical Trials Registry UMIN000026372; https://upload.umin.ac.jp/cgi-open-bin/ctr_e/ctr_view.cgi? recptno=R000030299 (Archived by WebCite at http://www.webcitation.org/743zBJj3D)

**International Registered Report Identifier (IRRID):**

DERR1-10.2196/12339

## Introduction

### Background

Stroke is a common disorder and one of the main causes of disability worldwide [1]. Although about 60% of stroke survivors reacquire the ability to walk independently, only 15% to 20% of them can use their affected upper limb practically [2-4]. Therefore, restoring the function of the paretic upper extremity is a challenging goal of rehabilitation.

Recent advances in neuroscience have shown that the adult human brain has a larger degree of plasticity to recover from neural damage than previously thought [5,6]. Clinically relevant interventional approaches to improve the paretic limb itself have been developed. A systematic review based on a meta-analysis of the effectiveness of neurorehabilitation approaches reported that constraint-induced movement therapy (CIMT), electromyographic biofeedback, mental practice with motor imagery, and robotic interventions are all favorable for recovery of arm motor function [7].

Although recent improvements in rehabilitation have succeeded in promoting functional recovery from poststroke hemiplegia, no effective interventions have been established for finger motor function [7]. The standardized mean difference in motor outcomes in the above-mentioned interventions is all around zero without statistical significance. Therefore, an important clinical challenge is development of a rehabilitation method for recovery of finger function.

Brain-machine interface (BMI) is a type of technology that can detect increased sensorimotor cortex excitability induced by a motor attempt of paretic finger extension. The sensorimotor rhythm (8-13 Hz) in electroencephalograms (EEGs) over the affected primary sensorimotor cortex decreases in amplitude because of desynchronization of oscillatory-coupled neural membrane potentials, called event-related desynchronization (ERD), when cortical excitability is increased. Therefore, EEG-ERD associated with an attempt of volitional movement of the paretic finger guarantees recruitment of the remaining sensorimotor cortical neurons, which are required for functional motor recovery. Somatosensory stimulation of the paretic finger, given through motor-driven hand orthosis and neuromuscular electrical stimulation (NEMS), is contingent on the occurrence of EEG-ERD and may allow sensorimotor coactivation that is restricted to the target corticomuscular region. This should help selective reinforcement of the targeted finger movement. Studies with healthy volunteers showed that motor imagery with BMI modulates intracortical inhibition of the primary motor cortex [8] and excitability of spinal anterior horn cells [9]. These findings suggested that BMI affects not only the sensorimotor cortex but also the entire corticospinal pathway.

Shindo et al reported that finger motor function of chronic, severe hemiparetic patients improves after 12 to 20 sessions of motor exercise with a BMI system for 1 hour [10]. Other studies with functional magnetic resonance imaging revealed that cortical activity of the affected sensorimotor cortex during execution of paretic finger movement is enhanced after BMI rehabilitation, whereas that of other regions, such as the unaffected sensorimotor cortex, is reduced [11,12]. These studies suggest that motor exercise with BMI promotes volitional recruitment of surviving motor pathways and facilitates paretic muscle activity. Such BMI-derived functional recovery is enhanced by combination with anodal transcranial direct current stimulation (tDCS), a known agent of increased neural plasticity of the central nervous system [13], suggesting that central nervous system neuroplastic changes may play a role in the process of BMI-derived functional recovery.

Improvement in the Fugl-Meyer assessment upper extremity motor function (FMA-UE) score was larger than minimum clinically important differences (MCID) of FMA-UE (4.25) [14] in both patients trained with BMI alone and patients trained with BMI with tDCS [13]. In a recent clinical study of patients with severe hemiparesis, improvement in FMA-UE was an average of 3.4 points after robot training [15]. Thus, BMI training can improve upper extremity function in patients with severe hemiparesis to a clinically meaningful level.

Japanese guidelines for the management of stroke 2015 (JGMS 2015) [16] and the American Heart Association/American Stroke Association (AHA/ASA) guideline [17] recommend certain rehabilitation techniques. CIMT improves upper extremity function [18] and is recommended by both JGMS 2015 [16] and the AHA/ASA guideline [17]. However, voluntary movement of fingers and the wrist is essential to perform CIMT, and whether CIMT has any advantage over dose-matched conventional rehabilitation is unclear. CIMT requires a well-trained therapist and high-dose training, which is 3 to 6 hours per day for 2 weeks in its original form.

NEMS is also recommended by the guidelines [16,17]. Electromyogram (EMG)-triggered NEMS is effective for patients with moderate hemiparesis who can voluntarily move their upper extremity [19]. In addition, hybrid assistive neuromuscular dynamic stimulation (HANDS) therapy is a combination of hand splints and 8-hour daily use of assistive NEMS, referred to as the integrated volitional electrical stimulator (IVES) with a hand splint. [20-22]. HANDS therapy can improve hand function in patients with more severe hemiparesis than what is approved for CIMT. However, HANDS therapy requires finger extensor EMG that can be detected by the IVES device. On the other hand, BMI rehabilitation can be used in patients who are not able to activate their paretic finger extensor at all. HANDS therapy following BMI training induces additional improvement in paretic upper limb function in patients who obtain improvement in extensor muscle activities with BMI training [22].

The repetitive facilitative exercise (RFE) program is a new rehabilitation method that is a combination of high-frequency repetitive voluntary movements and neurofacilitation [23]. RFE improves the function of paretic upper extremities in subacute stroke patients. However, RFE can be used in patients with only mild to moderate hemiparesis, and special skills are required.

Noninvasive brain stimulation methods, such as repetitive transcranial magnetic stimulation and tDCS, are used in patients with mild to moderate hemiparesis, and the effectiveness of the combination of brain stimulation and intensive upper limb rehabilitation has been reported in several studies [24-26]. However, no methods have been established for use of brain stimulation of hemiplegic patients [25], and application of these methods in poststroke patients requires safety considerations [27,28].

Robotic therapy is recommended for consideration in patients with moderate to severe hemiparesis according to the AHA/ASA guideline [17]. A systematic review found that robotic therapy improves arm function [29]. However, whether robotic therapy is more effective than dose-matched conventional upper limb exercise therapies is uncertain [17].

### Study Objectives

A systematic review and several guidelines [7,16,17] showed that some types of interventions (such as CIMT, EMG biofeedback, NEMS, mental practice, and robotics) can improve paretic arm function. However, no rehabilitation method has been verified to be effective for improving paretic hand function. Therefore, we planned a randomized controlled trial (RCT) to evaluate the effectiveness and safety of an intensive rehabilitation program using the EEG-based BMI rehabilitation system for patients with severe hemiparetic stroke.

## Methods

### Study Design

This is a single-blinded, multicenter RCT using a parallel arm design to evaluate the effectiveness and safety of 2-week BMI rehabilitation combined with intensive occupational therapy compared with motor imagery without any feedback and dose-matched occupational therapy. Overall, 4 hospitals in Japan participated in this trial. Patients were randomly assigned to either the BMI group or control group. The assessors were blinded, but patients were not. The primary outcome measure was change in upper extremity score of FMA between baseline and 4 weeks after the intervention.

### Participants and Recruitment

The inclusion criteria were (1) time from stroke onset to be more than 90 days; (2) first ever stroke patients with upper extremity paresis; (3) no loss of proprioception in paretic fingers (patients able to detect a position change after maximum possible motion); (4) ability to raise the paretic hand to the height of the nipple; (5) passive range of motion greater than −10 degrees for metacarpophalangeal joint extension; (6) ability to flex the paretic fingers voluntarily but not to extend them; (7) ability to walk independently in daily life with or without assistance; (8) ability of the patient to understand and consent to the study protocol; and (9) aged 18 years or older at the time of agreement to participate in this study. The exclusion criteria were (1) serious medical conditions that would interfere with rehabilitation such as severe heart disease, uncontrolled hypertension, history of pulmonary embolism, acute pulmonary heart disease or severe pulmonary hypertension within 90 days before enrollment, severe hepatic or renal dysfunction, severe orthopedic impairment, severe cognitive or psychiatric disorder, and other serious medical conditions; (2) pacemaker or use of other implanted stimulators; (3) history of seizures within 90 days before enrollment; (4) participation in another clinical trial for regulatory approval within 90 days before enrollment; (5) receiving other special neurorehabilitation techniques for upper extremity paresis such as transcranial magnetic stimulation, therapeutic electrical stimulation, CIMT, and repetitive facilitative exercise within 90 days before enrollment; (6) injection of botulinum toxin or phenol for treatment of upper-limb spasticity within 90 days before enrollment; (7) impossible to record EEG because of skin status or skull deformity; or (8) other critical problems that would affect participation in the study.

Prospective participants were recruited from outpatients of the rehabilitation department in Keio University Hospital, Saiseikai Kanagawa-ken Hospital, Tokyo Metropolitan Rehabilitation Hospital, and Tokyo Bay Rehabilitation Hospital. The recruiting physiatrists screened the participants for eligibility. We obtained written informed consent from patients who met all inclusion criteria and did not meet any exclusion criteria except exclusion criterion 7. The patients then tried the BMI system to check the skin status and rule out a skull deformity. If EEG could be recorded, the patient was registered as an eligible and consenting participant. Then they completed the baseline assessment. Treatment started within 28 days after registration.

### Study Procedures

**Figure 1:**
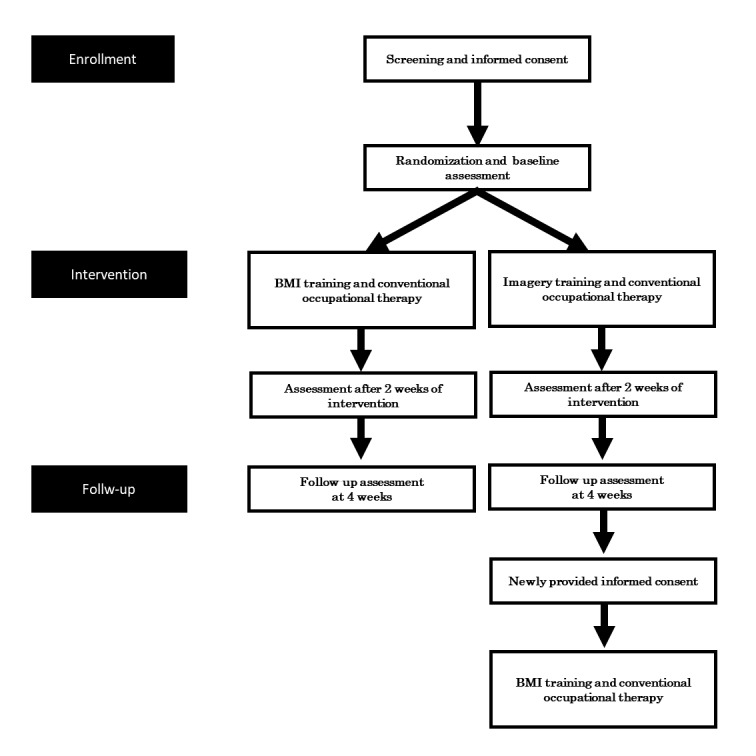
Study design. BMI: brain-machine interface.

Study procedures are summarized in the Consolidated Standards of Reporting Trials diagram (Figure 1).

After follow-up assessment, participants allocated to control group received the same BMI training that would be conducted for participants allocated to BMI group if they wished and had newly provided informed consent. This BMI training started within 60 days after follow-up assessment.

### Randomization

Participants were randomly allocated to the BMI or the control group using a computerized block randomization scheme, including prestratification according to each participating hospital.

### Blinding

The rater was blinded to treatment allocation, and the rater was not involved in the participants’ treatment. The blinded rater assessed FMA [30-36]. Rater blinding was verified with a yes or no question: Did you rate the assessment score blindly? Participants were not blinded to their own treatment.

### Intervention

#### Brain-Machine Interface Training

##### Electroencephalogram Recording

An Ag-AgCl electrode (φ=9 mm) for EEG measurement was placed over the ipsi-lesional sensorimotor cortex, namely, C3 (for the left hemisphere) or C4 (for the right hemisphere) according the international 10-20 system. An additional electrode was placed 20 mm lateral to C3 or C4. A ground electrode was placed on A1, and the reference electrode was placed on A2 (ipsilateral to the lesioned hemisphere). All electrodes were guided manually and fixed with a custom-made headset. The application-specific integrated circuit–based analog circuit and microprocessor were embedded inside the headset, and 2-channel EEGs were derived in a monopolar manner and processed with ×1200 amplification and 0.21- to 199-Hz filtering. The processed EEG signals were digitized at 200 Hz with 12 bits (least significant bit 0.366 μV). Note that a notch filter of 50 Hz was used to minimize the power-line noise. EEGs were then transmitted to a laptop using a Bluetooth 3.0 wireless protocol and were subtracted from each other to derive a bipolar EEG. A 2 Hz to 50 Hz bandpass filter with a 50 Hz notch was again used to reduce noise contamination.

A 1-second time-sliding Hanning window was applied to this bipolar EEG signal with 87.5% overlap, and fast Fourier transform was applied to obtain the time-varying power spectrum of the signal. The two-dimensional (2D) feature vector with mean alpha frequency band power (7-13 Hz) and mean beta frequency band power (14-26 Hz) was constructed at each time segment and traced with time in the feature space. The discriminant line that determines EEG feature vectors as in either the *ERD* or *baseline* class was used for EEG labeling. The discriminant line was calibrated for each participant every day before the training session (see also Calibration section below).

##### Calibration

At the beginning of the BMI training, 10 trials of the cue-based motor task were conducted as a rehearsal, and the parameters in the linear discriminant analysis (LDA) for EEG-ERD detection in the BMI were calibrated using the obtained data. During the rehearsal, a 5-second resting period was first given and a text cue of either “attempt paretic finger extension” or “keep relaxing” was then displayed on the top of the computer screen. The participants performed the given cued task for the next 5 seconds. In total, 10 trials per class were given in a randomized order. A 2D feature vector with mean alpha frequency band power (7-13 Hz) and mean beta frequency band power (14-26 Hz) was obtained from a 1-second time-sliding Hanning window with 87.5% overlap. The feature vectors with annotations of either “attempt paretic finger extension” or “keep relaxing” were mapped onto the feature space, and the parameters in the LDA algorithm were optimized to separate the features into appropriate classes. Consequently, the LDA in the BMI training returned a value of *+1* (resting) or *−1* (finger extension) every 125 ms according to the EEGs.

##### Estimation of Sensorimotor Cortex Excitability From Electroencephalogram

Alpha and beta frequency band powers in EEG recorded over the sensorimotor cortex are analogs of sensorimotor cortex excitability [37]. EEG-ERD of these powers is also correlated with corticospinal tract excitability, disinhibition of gamma-aminobutyric acid-ergic intracortical inhibitory circuits [8], and spinal anterior horn cell excitability [9]. Extrapolation of these findings to poststroke patients with hemiplegia may be acceptable because ERD during paretic hand motor imagery is associated with ipsi-lesional corticospinal tract excitability in poststroke patients with hemiplegia [38]. Alpha frequency oscillation and its resonance among cortical and subcortical regions in poststroke patients with hemiplegia predicted motor outcome, suggesting that the alpha component is related to sensorimotor function. Recent clinical studies with BMI intervention also suggest that up-conditioning of EEG alpha and beta band frequency powers and their ERD during motor attempting of paretic finger opening through BMI training is associated with increased corticospinal tract excitability [10] and the blood oxygen level-dependent signal of magnetic resonance imaging in the ipsi-lesional sensorimotor cortex [11,12]. Repeated use of BMI that forces patients to increase alpha and beta frequency powers at rest and decrease their power during paretic hand motor attempting is, therefore, interpreted as neurorehabilitative training of the remaining corticomuscular pathway on the ipsi-lesional side.

##### Task-Specific Brain-Machine Interface Training

BMI training was conducted for approximately 40 min per session, 1 session per day, and 5 days per week, for 2 weeks.

The motor-driven orthosis was attached to the affected hand to achieve finger extension movement at the metacarpophalangeal and proximal interphalangeal joints. The orthosis was designed to remedy the finger position as the fingers arched with the thumb opposed, helping patients to hold or release objects. The thumb opposition position also helps maintain decreased flexor spasticity. The range of the angle in the orthosis action was adjusted by the examiner to avoid pain and spasticity. During the training, the motor-driven orthosis helped finger extension by a predetermined angle with 1659 Nmm at maximal torque. This setting allowed patients to perform task-specific training with BMI in a real-world setting.

A pair of disposable electrodes (25 × 45 mm) for electrical stimulation was placed over the belly of the paretic extensor digitorum communis muscle. Test stimulation (biphasic rectangular wave, pulse width of 1 ms, and frequency of 100 Hz) was then given, and the intensity was set at just above the motor threshold. Note here that this is a known intensity for recruiting Ia proprioceptive afferents of the stimulating muscle. Such NEMS of the target muscle of motor imagery contingent upon the occurrence of ERD may allow sensorimotor coactivation to be restricted to the target corticomuscular region and may foster plasticity and motor learning [39]. This should further help to selectively reinforce the sensorimotor representations.

The affected forearm was set on a balanced forearm orthosis. The participants sat in front of the desk, and 30 pegs were set on the desk peg board. Participants were asked to pick up a peg with the affected hand with the orthosis. After pinching a peg with the affected hand, participants pressed a button to start the preset BMI training sequence. As in the calibration session, 5 seconds of rest were first given, and a text cue of either “attempt paretic finger extension” or “keep relaxing” was then displayed on the top of the computer screen. The participants performed the given cued task for the next 5 seconds. The LDA-based EEG classifier returns either the value *+1* (EEG was at the resting condition) or *−1* (EEG-ERD) every 125 ms according to the EEGs and triggers the motor action of the orthosis and NEMS if a successive 1 second of the class *−1* is given in either cue of “attempt paretic finger extension” or “keep relaxing.” Note here that EEG-ERD-associated sensorimotor stimulation via the orthosis and electrical stimulation during attempting paretic finger extension function as positive reinforcers of the training. Patients tried to increase the probability of this condition through trial and error. The motor-driven orthosis and NEMS were not activated if EEG did not satisfy the criteria (even if participants attempted finger opening). Repeated use of EEG-ERD-based BMI can, therefore, be interpreted as a reinforcer of the remaining corticomuscular pathway on the ipsi-lesional side.

##### Motor Imagery Training in the Control Group

Participants in the control group conducted the motor imagery training for approximately 40 min for 2 weeks, similar to the BMI training for participants in the BMI group. Participants wore the same headset as those in the BMI training, but they did not wear the hand orthosis. They were instructed to rest for 5 seconds and then to imagine extending their affected fingers for the next 5 seconds in the same manner, as those in the BMI training. At that time, the EEG was recorded in the same way as during the BMI training, but no feedback was given to the participants about the quality of the EEG. Neither passive movement or electrical stimulation was given during imagery.

##### Conventional Occupational Therapy

All participants received 40 min of standard occupational therapy per day, which consisted of gentle stretching exercises, active muscle re-education exercises, and introduction to bimanual activities in their daily lives.

#### Concomitant Care and Recommendation

During the 6-week period of intervention and follow-up, participants were asked not to undertake other specific intervention intending to improve hemiparesis (eg, CI therapy, NEMS, robotic rehabilitation, noninvasive brain stimulation) and botulinum toxin injection to hemiparetic upper limb. Moreover, they were asked not to change dose of antiepileptic agents, muscle relaxants, psychotropic agents, and anxiolytic agents. If participants were undertaking conventional occupational therapy before participating in this study, they were asked not to exceed the time and frequency of it during 30 days before intervention.

#### Intervention Fidelity and Monitoring of Adverse Events

Before beginning this study, treatment therapists were trained by a member of the research team with a high-level experience in BMI training for stroke patients. During the whole duration of the study, the members of the research team and research coordinator randomly visited training sessions, to ensure that scheduled intervention was being performed accurately and with high adherence to the protocol proposed. Any adverse unpredictable event was recorded in the registry of each patient and the electronic database of the study and managed according to the policies of the hospital, with referral appropriate medical follow-up.

#### Criteria for Withdrawal

Participants were withdrawn from the study in the event of any relevant deterioration in health likely to affect participation or if they withdrew their consent.

#### Outcome Measurement

#### Schedule of Assessment

The primary outcome measure was assessed by a blinded evaluator. Other functional measurements were assessed by evaluators who were trained by the organizer of this RCT. Most assessments were conducted at baseline, after intervention (post), and 4 weeks after intervention (follow-up). EEGs were recorded by the EEG-BMI rehabilitation system during each training session. The schedule of assessments is shown in Table 1.

**Table 1:** Schedule of assessments.

Time points and measure		Baseline	Intervention (10 sessions during 2 weeks)	Posttreatment	Follow-up (4 weeks after treatment)
**Primary outcome measure**					
	Fugl-Meyer assessment	B^a^	—^b^	B	B
**Secondary outcome measure**					
	Action Research Arm Test	E^c^	—	E	E
	Motor Activity Log-14	E	—	E	E
	Stroke Impairment Assessment Set	E	—	E	E
	Modified Ashworth Scale	E	—	E	E
	Barthel Index	E	—	—	E
	Goal Attainment Scale	S^d^	—	—	S
	Stroke-Specific Quality of Life Scale	S	—	—	S
	Surface electromyography	EP^e^	—	EP	EP
	Electroencephalography during brain-machine interface or imagery training	—	EP	—	—

^a^B: assessment of blinded evaluator.

^b^Dashes indicate "not applicable."

^c^E: assessment of well-trained evaluator.

^d^S: participant self-report.

^e^EP: electrophysiological data.

#### Primary Outcome Measure

Fugl-Meyer assessment Upper extremity motor function was assessed with the FMA (range 0-66 points, total score) [30,31]. FMA consisted of test A (shoulder/elbow/forearm: 36 points, A score), test B (wrist: 10 points, B score), test C (hand/finger: 14 points, C score), and test D (coordination: 6 points, D score). FMA was assessed according to the scoring manual [32], and the validity and reliability were previously confirmed [31,33].

The estimated clinically important difference of the FMA-UE scores ranged from 4.25 to 7.25 points in individuals with stable, mild to moderate upper extremity hemiparesis [34]. However, MCID for patients with severe hemiparesis remains to be shown. As a greater than 10% change in FMA motor scores may represent a clinically meaningful improvement based on clinical experience [35], MCID for severe hemiparesis may be lower than that for mild hemiparesis. A minimal detectable change of 3.2 points was reported in 31 patients with stroke [36].

#### Secondary Outcome Measures

##### Action Research Arm Test

Action Research Arm Test (ARAT) [40] is a frequently used, validated, and reliable measure of upper extremity function with 4 subsections: grip, grasp, pinch, and gross movement [41,42]. The maximum summed score is 57.

##### Motor Activity Log-14

Upper extremity disability in activities of daily living (ADL) was assessed with Motor Activity Log (MAL), which uses a structured interview [43]. MAL includes 14 items, scored on an 11-point amount of use scale (range 0-5) to rate how much the arm is used (MAL-amount of use) and an 11-point quality of movement scale (range 0-5) to rate how well the participants are using their affected upper extremity [43]. High construct validity and reliability have been reported in patients with chronic stroke [43,44].

##### Motor Scores of the Stroke Impairment Assessment Set

The Stroke Impairment Assessment Set (SIAS) is a comprehensive instrument for assessing stroke impairment with well-established psychometric properties [45,46]. SIAS assesses various aspects of impairment in stroke patients, including motor function, tone, sensory function, range of motion, pain, trunk function, visuospatial function, speech, and sound side function. Motor scores of the SIAS are composed of 5 items that assess arm, finger, hip, knee, and ankle functions and are rated from 0 (severely impaired) to 5 (normal).

##### Goal Attainment Scale

The Goal Attainment Scale (GAS) is a self-rating scale to evaluate subjective improvement following rehabilitation [47-50]. Patients rate the attainment level of the rehabilitation outcome for the goal that they set themselves. If the attainment is as expected, it is rated as 0. Improvement beyond expectation is rated +1 or +2 and that below expectation is rated −1 or −2.

##### Barthel Index

The Barthel Index (BI) is one of the most frequently used measures to evaluate ADL in stroke research [51,52]. BI measures independence in ADL; the maximum score is 100. The 10 assessed items of ADL are feeding, bathing, grooming, dressing, bowel control, bladder control, toilet use, transfers, mobility, and ascending and descending stairs.

##### Modified Ashworth Scale

Spasticity at the wrist and finger flexors of the affected upper extremity was assessed with the Modified Ashworth Scale (MAS), a 6-point rating scale used to measure passive muscle resistance [53].

##### Stroke-Specific Quality of Life Scale

The Stroke-Specific Quality of Life Scale (SS-QOL) was developed to assess health-related quality of life in stroke patients [54,55]. SS-QOL contains 49 items and covers 12 different areas of quality of life affected by stroke. The 12 areas of the SS-QOL are energy, family roles, language, mobility, mood, personality, self-care, social roles, thinking, upper extremity function, vision, and work or productivity. Each area can be scored separately, but a total score is also available. The possible range of all scales is from 1 to 5, where a lower value indicates a lower health-related quality of life.

##### Surface Electromyography

The muscle activities of the paretic extensor digitorum communis and flexor digitorum superficialis muscles were recorded with Ag-AgCl surface electrodes with diameters of 9 mm (Nihon Kohden, Tokyo, Japan). The electrodes were applied with center-to-center spacing of 30 mm and were placed parallel to the muscle fibers and distal from the motor points of individual muscles. Before the electrodes were attached, the skin areas were rubbed with alcohol. Skin resistance was kept below 5 kΩ. An MEB-2300 EMG machine (Nihon Kohden) was used to record and analyze the EMG data. The bandpass filter was set at 20 Hz to 1 kHz. The patients were seated in a comfortable chair with their arms on an armrest and the angle of their elbows was kept at 70 to 90 degrees. They were instructed to rest for 5 seconds and then to extend their affected fingers for the next 5 seconds for 1 cycle. In total, 5 cycles of 5 seconds of rest and 5 seconds of extension were repeated.

##### Electroencephalogram During Brain-Machine Interface or Image Training

The EEGs during BMI or image training were recorded and stored in the EEG-BMI system. We compared the magnitude and duration of μERD between the BMI group and control group.

#### Statistical Analyses

The following 2 analysis populations are defined in this clinical trial. Statistical analyses were performed for each patient population.

The Full Analysis Set (FAS) essentially included all randomized patients, in accordance with the intention-to-treat principle, except for the following patients who violated major conditions in this study:

- Patients who did not meet major inclusion criteria in this trial
- Patients who did not receive any study treatment
- Patients who had no baseline measurements or no measurements after baseline
- Patients who withdrew informed consent after randomization and refused to generate any data in this trial

The Per Protocol Set (PPS) included patients who were compliant with the protocol and are defined in the patient-data handling document that will be finalized before the unblinding of this trial.

The primary analysis population in this trial is the FAS. The primary endpoint, a change from baseline in FMA at 28 days after study treatment, will also be analyzed for the PPS. The sensitivity of the primary analysis results will be examined via a comparison of results between the 2 analysis sets. All patients who received at least one study treatment are included in the safety analysis. The patient-data handling document, including the data handling rule for measurement at each time point, will be finalized before the unblinding. Methods to handle missing data will also be included in the statistical analysis plan (SAP) for this trial. Demographic factors and baseline characteristics are summarized by the treatment group.

#### Efficacy Analysis

##### Analysis of the Primary Endpoint

The primary endpoint of this trial is a change from baseline in the FMA at 28 days after study treatment. The superiority of the BMI group compared with the control group in terms of the primary endpoint will be demonstrated by means of an analysis of covariance model, which contains the baseline as a covariate and treatment group as a factor. Least squares means for the changes in treatment groups will be estimated and compared between the 2 groups. Missing data at 28 days after study treatment will be handled by multiple imputation, Rubin, which assumes missing at random as the missing data mechanism [56]. The details of the imputation model in the multiple imputation will be described in the SAP before the unblinding.

##### Analysis of Secondary Endpoints

For continuous endpoints, the same analysis model will be applied. For binary endpoints, 95% CIs for proportions by treatment groups will be estimated with the Clopper-Pearson method. The proportions of the 2 groups will be compared with Fisher exact test.

#### Safety Analysis

##### Adverse Events and Device-Related Problems

The safety primary endpoints in this study are proportions of adverse events and device-related problems. The number of events and the proportion will be calculated, and 95% CIs for the proportions will be estimated with the Clopper-Pearson method.

#### Sample Size Estimation

The total sample size in this trial was set at 40 patients (20 per group).

##### Rationale

The mean changes from baseline in the FMA at 28 days after study treatment are assumed to be 6.9 and 3.0 in the BMI and control groups, respectively, based on previous clinical studies [57,58]. The SD for the change is also assumed to be 4.0. Under these assumptions, 17 patients per group results in 80% power for testing the between-group difference in means with a two-sided 5% alpha. Taking into account 15% exclusions from the analysis, the target sample size in this trial was set at 20 patients per group (total of 40 patients).

#### Significance Level and Multiplicity

Significance levels for all tests in this trial are set at two-sided 5%, and confidence levels for all interval estimations are two-sided 95%. No multiplicity adjustment will be performed because this study has a single efficacy primary endpoint.

#### Interim Analysis

No interim analysis was conducted in this trial.

#### Change in Original Statistical Analysis Plan

All changes in the original SAP will be reported in the clinical study report for this trial.

#### Statistical Analysis Plan

The SAP for this study, which contains comprehensive details of data handling and statistical analysis methods, will be finalized before unblinding of this study.

### Ethics and Dissemination

This study was conducted in accordance with Ministerial Ordinance on Good Clinical Practice for Medical Devices [59]. All participants provided voluntary written informed consent. Prospective participants were fully informed about what study participation involved and the potential benefits and risks. Ethics approvals had been obtained from the institutional review board of Keio University (protocol number: KCTR-D008, reference number: D16-03). In addition, for Saiseikai Kanagawa-ken Hospital, Tokyo Metropolitan Rehabilitation Hospital, and Tokyo Bay Rehabilitation Hospital, ethics approval had been obtained from the institutional review board of Saiseikai Kanagawa-ken Hospital (protocol number: KCTR-D008). Any protocol amendments will be submitted for ethical approval and communicated to the trial registry. The results of this study will be used to gain regulatory approval for the manufacture and sale of the medical device used in this study in Japan. After approval, the results will be presented at scientific meetings and published in journals.

## Results

Recruitment started in March 2017 and ended in July 2018. This trial is currently in the data correcting phase. This RCT is expected to be completed by October 31, 2018.

## Discussion

This multicenter RCT was designed to demonstrate the effect of EEG-BMI training compared with simple mental imagery on upper extremity function, including finger function, in poststroke patients with severe impairment of finger function. Although several neurorehabilitation approaches have shown clinically important improvements in arm function of poststroke, hemiparetic patients, no intervention is widely accepted as effective treatment for improving finger function [7,16-18]. If this device is approved for manufacturing and sale by the Japanese government, it will be the first neurorehabilitation product targeting improvement of finger function. Clinically and scientifically, the results of this RCT will provide significant knowledge about the contribution of sensorimotor feedback to motor learning and recovery in poststroke patients compared with open-loop imagery.

Several studies have reported the effectiveness of rehabilitation using other BMI systems for chronic stroke patients with upper extremity paresis [58,60-63]. However, no device is approved as an official medical device. In addition, this trial will demonstrate that closed-loop sensory-motor feedback training with the EEG-BMI rehabilitation system can induce functional recovery and that this improvement will be maintained or will increase 4 weeks after the intervention, because the primary outcome is the change in FMA between baseline and follow-up 4 weeks after the end of the intervention. If rehabilitation induces plastic changes in the brain of chronic stroke patients, acquired recovery will be sustained or will increase in the several weeks following the intervention. On the other hand, the recovery will decrease if the effect of rehabilitation is transient. In this study, the control intervention is based on open-loop motor imagery training without any feedback. Therefore, the results of this RCT will provide several important suggestions about the mechanisms of the effect of closed-loop sensory-motor feedback training on neural plasticity in the damaged brain by comparing closed-loop feedback training using the EEG-BMI system with dose-matched, open-loop motor imagery training. This is a unique scientific point of view in this RCT compared with other studies of BMI rehabilitation.

In this RCT, we assess a sufficient number of functional measures of the upper extremity (such as ARAT, MAL, motor score of SIAS, and MAS), subjective satisfaction with the treatment (GAS), ADL (BI), and QOL (SS-QOL) to make the outcome of this RCT comparable with those of other trials. In addition, electrophysiological assessments (surface EMG and EEG during BMI training) will be adopted to elucidate the underlying mechanisms of improvement with BMI rehabilitation.

## Appendix

Multimedia Appendix 1 Peer-review report from the Japan Agency for Medical Research and Development (Part 1).

Multimedia Appendix 2 Peer-review report from the Japan Agency for Medical Research and Development (Part 2).

Multimedia Appendix 3 Translation of the peer-review report from the Japan Agency for Medical Research and Development (Part 1).

Multimedia Appendix 4 Translation of the peer-review report from the Japan Agency for Medical Research and Development (Part 2).

## Abbreviations

ADL: activities of daily living
AHA: American Heart Association
AMED: Agency for Medical Research and Development
ARAT: Action Research Arm Test
ASA: American Stroke Association
BI: Barthel Index
BMI: brain-machine interface
CIMT: constraint-induced movement therapy
EEG: electroencephalogram
EMG: electromyography
ERD: event-related desynchronization
FAS: full analysis set
FMA: Fugl-Meyer assessment
GAS: Goal Attainment Scale
HANDS: hybrid assistive neuromuscular dynamic stimulation
IVES: integrated volitional electrical stimulator
JGM 2015: Japanese guidelines for the management of stroke 2015
LDA: linear discriminant analysis
MAL: Motor Activity Log-14
MAS: Modified Ashworth scale
MCID: minimal clinically important difference
NEMS: neuromuscular electrical stimulation
PPS: per protocol set
RCT: randomized controlled trial
RFE: repetitive facilitative exercise
SAP: Statistical analysis plan
SIAS: Stroke Impairment Assessment Set
SS-QOL: Stroke-Specific Quality of Life Scale
tDCS: Transcranial direct current stimulation
2D: two-dimensional
UE: upper extremity
