# Supplementary material for: Evaluating the Effectiveness and Safety of the Electroencephalogram-Based Brain-Machine Interface Rehabilitation System for Patients With Severe Hemiparetic Stroke: Protocol for a Randomized Controlled Trial (BEST-BRAIN Trial)
Source: JMIR Res Protoc. 2018 Dec 6;7(12):e12339. doi: 10.2196/12339 (PMC6302229; doi:10.2196/12339)
Supplement: Multimedia Appendix 1 [file resprot_v7i12e12339_app1.pdf]

27医研開第3283号
平成２８年３月８日

慶應義塾大学
教授
里宇 明元　殿

国立研究開発法人　日本医療研究開発機構
理事長　　　　末松　誠　　　　印

「医療機器開発推進研究事業平成28年度1次公募」に係る
選考結果について

拝啓、　時下ますます御清栄のこととお喜び申し上げます。
さて、先般公募いたしました標記事業につきまして、御応募をいただき厚くお礼申し上げます。
応募のありました貴提案について、当機構において有識者による厳正な審査を行った結果、貴提案を別紙に示す通り、条件付採択することに決定いたしましたので、通知いたします。
今後とも当機構の実施する事業につきまして、ご支援、ご協力を賜りますようお願い申し上げます。

敬具

【受理番号】3-06
【提案名】「脳卒中後上肢麻痺に対する脳波―BMIリハビリテーションシステムの医師主導治験」

別紙１

理由書

【提案】
受理番号　3-06
脳卒中後上肢麻痺に対する脳波―BMI リハビリテーションシステムの医師主導治験

【提案者名】
慶應義塾大学

【審査結果】
条件付採択

【理由】
ご提案内容を公募要領に示した審査基準に基づき、厳正かつ慎重に審査しました。
貴提案については、BMI リハビリシステムの実用化へ向けた着実な研究・開発実績をもとにした革新的医療技術・機器の開発と企業への導出が期待できることが評価されました。

【採択条件】
- 導出先であるパナソニック(株)エコソリューション社は製造販売業を持っていないので、製品化に向けて、平成 28 年度内に業許可等の方針を決めること。
- 到達目標について、AMED と相談し、より定量的な中間・最終目標を設定すること。
- AMED が提案した予算額で、事業の主旨及び提案内容を損なうことなく、適切に実施すること。
