# Supplementary material for: Evaluating the Effectiveness and Safety of the Electroencephalogram-Based Brain-Machine Interface Rehabilitation System for Patients With Severe Hemiparetic Stroke: Protocol for a Randomized Controlled Trial (BEST-BRAIN Trial)
Source: JMIR Res Protoc. 2018 Dec 6;7(12):e12339. doi: 10.2196/12339 (PMC6302229; doi:10.2196/12339)
Supplement: Multimedia Appendix 2 [file resprot_v7i12e12339_app2.pdf]

16hk0102032h0001
29医研開第2636号
平成２９年９月１２日

学校法人慶應義塾
理事長　長谷山　彰
上記代理人　慶應義塾大学医学部長
岡野　栄之　殿

国立研究開発法人日本医療研究開発機構
契約担当職　理事長　末松　誠

確　定　通　知　書

平成２８年度委託研究開発契約に基づく確定検査の結果、下記のとおり額の確定をしたので通知します。

記

1．事　業　名　医療機器開発推進研究事業/

2．研究開発課題名　脳卒中後上肢麻痺に対する脳波-BMIリハビリテーションシステムの医師主導治験

3．研究開発担当者　所属　役職　氏名　医学部　教授　里宇　明元

4．検　査　日　平成29年9月4日

5．委託研究開発費　56,800,000円

6．確　定　額　56,800,000円

7．既支払済額　56,800,000円

8．返　還　額　0円

以　上
