# Supplementary material for: Evaluating the Effectiveness and Safety of the Electroencephalogram-Based Brain-Machine Interface Rehabilitation System for Patients With Severe Hemiparetic Stroke: Protocol for a Randomized Controlled Trial (BEST-BRAIN Trial)
Source: JMIR Res Protoc. 2018 Dec 6;7(12):e12339. doi: 10.2196/12339 (PMC6302229; doi:10.2196/12339)
Supplement: Multimedia Appendix 4 [file resprot_v7i12e12339_app4.pdf]

16hk0102032h0001
29AMED No. 2636
September 12, 2017

To: Akira Hasegawa
Dean, Keio University School of Medicine,
Hideyuki Okano
Representative of Akira Hasegawa, President of Keio University School of Medicine

From: Makoto Suematsu,
President, in charge of contract, Japan Agency for Medical Research and Development

Notice of Determination

This is a notice of the amount of the grant determined by the result of review based on the contract for granted research and development in fiscal year 2016.

1. Business name: Research on Development of New Medical Devices
2. Title of research and development program: A clinical trial for regulatory approval to evaluate the effectiveness and safety of an electroencephalography-based brain-machine interface rehabilitation system for patients with severe hemiparetic stroke
3. Affiliation, title, name of the Person responsible for research and development: Meigen Liu, Professor, School of Medicine
4. Date of review: September 4, 2017
5. Amount of grant for research and development:
6. Determined amount: 56,800,000 yen
7. Paid amount: 56,800,000 yen
8. Refunded amount: 0 yen
