# Supplementary material for: Evaluating the Effectiveness and Safety of the Electroencephalogram-Based Brain-Machine Interface Rehabilitation System for Patients With Severe Hemiparetic Stroke: Protocol for a Randomized Controlled Trial (BEST-BRAIN Trial)
Source: JMIR Res Protoc. 2018 Dec 6;7(12):e12339. doi: 10.2196/12339 (PMC6302229; doi:10.2196/12339)
Supplement: Multimedia Appendix 3 [file resprot_v7i12e12339_app3.pdf]

27 AMED No. 3283

March 8, 2016

To: Department of Rehabilitation Medicine Keio University School of Medicine,
Professor,
Meigen Liu

From: Makoto Suematsu,
President, in charge of contract, Japan Agency for Medical Research and Development

Notice of results of screening for the first call for proposals of FY2016 in the Medical Device Development Promotion Research Program

Dear Professor Liu,

Greetings and best wishes.

Thank you for submitting your proposal to the Medical Device Development Promotion Research Program.

This is to notify you that your proposal has been selected for conditional acceptance following careful screening by experts affiliated with our Organization, as described on the attached sheet.

We hope you continue cooperation and support of our activities.

Sincerely yours,

【Receipt number】3-06

【Title】Investigator initiated clinical trial to evaluate efficacy and safety of brain wave – BMI rehabilitation system for the hemiplegic upper limb

Attachment 1

Reasons for screening results

Proposal<br>Receipt number: 3-06<br>Title: Investigator initiated clinical trial to evaluate efficacy and safety of brain wave – BMI rehabilitation system for the hemiplegic upper limb
Institution Name<br>Keio University
Results of screening:<br>Conditional acceptance
Reasons:<br>The content of your proposal has been reviewed strictly and carefully based on the screening standards described in the call for proposals.<br>Your proposal has been assessed to have promise in the areas of development of innovative technologies and devices and corporate business development based thereon, grounded in the results of steady research and development toward practical implementation of BMI rehabilitation systems.<br><br>Conditions of acceptance:<br>- Since the Panasonic Eco Solutions Company, which plans to conduct business development based on this technology, does not engage in the businesses of manufacturing and sales, we ask you decide on policies for obtaining the necessary business permits etc. during FY2016 in preparation for product development.<br>- Consult with AMED to decide on quantitative interim and final objectives.<br>- Implement the project appropriately within the budget amount proposed by AMED, without detracting from the purposes of the program or the content of the proposal.
